# Ethics teaching in a medical education environment: preferences for diversity of learning and assessment methods

**DOI:** 10.1080/10872981.2017.1328257

**Published:** 2017-05-31

**Authors:** Tahra AlMahmoud, M. Jawad Hashim, Margaret Ann Elzubeir, Frank Branicki

**Affiliations:** ^a^ Department of Surgery, College of Medicine and Health Sciences, United Arab Emirates University, Al Ain, United Arab Emirates; ^b^ Department of Family Medicine, College of Medicine and Health Sciences, United Arab Emirates University, Al Ain, United Arab Emirates; ^c^ Department of Medical Education, College of Medicine and Health Sciences, United Arab Emirates University, Al Ain, United Arab Emirates

**Keywords:** Medical ethics, professionalism, teaching and learning

## Abstract

**Background**: Ethics and professionalism are an integral part of medical school curricula; however, medical students’ views on these topics have not been assessed in many countries.

**Objective: **The study aimed to examine medical students’ perceptions toward ethics and professionalism teaching, and its learning and assessment methods.

**Design**: A self-administered questionnaire eliciting views on professionalism and ethics education was distributed to a total of 128 final-year medical students.

**Results**: A total of 108 students completed the survey, with an 84% response rate. Medical students reported frequently encountering ethical conflicts during training but stated only a moderate level of ethics training at medical school (mean = 5.14 ± 1.8). They noted that their education had helped somewhat to deal with ethical conflicts (mean = 5.39 ± 2.0). Students strongly affirmed the importance of ethics education (mean = 7.63 ± 1.03) and endorsed the value of positive role models (mean = 7.45 ± 1.5) as the preferred learning method. The cohort voiced interest in direct faculty supervision as an approach to assessment of knowledge and skills (mean = 7.62 ± 1.26). Female students perceived greater need for more ethics education compared to males (p = < 0.05). Students who claimed that they had experienced some unprofessional treatment had a more limited view of the importance of ethics as a subject (P = 0.001).

**Conclusion**: Medical students viewed ethics education positively and preferred clinically attuned methods for learning.

## Introduction

Whereas knowledge and competencies are the primary goals of formal medical training, an understanding of professional values and ethical conduct is essential for fostering the development of a good doctor [1–3]. In recent years, medical ethics has become a universal component of undergraduate and graduate education and clinical training [4]. Yet, a unified theoretical or practical model to integrate the teaching of professionalism into curricula does not exist [5]. Furthermore, ethics curricula have often been structured in relation to abstract bioethical principles rather than considerations of context, trainees’ experiences, and self-identified educational needs [6]. This is despite the fact that over the last three decades several studies have shown that a majority of medical students (64–84%) believe that ethical practices are critically important in the provision of the highest standards of medical care [7,8]. Students have also expressed pronounced enthusiasm (up to 95%) to learn more about medical ethics [7–9]. By understanding how students learn medical ethics we can plan the most effective ways in which to help them learn. In an outcomes-based curriculum experiential, adult learning, social constructivist and reflective theories [10–13] represent important theoretical frameworks underpinning learning strategies suited to medical ethics teaching and learning; since to develop ethics and professional behaviors all learners should not only identify their learning needs, gaps in knowledge, and understanding but also be able to observe the performance of role models, discuss and make sense of problems, practice application of skills in classroom and clinical environments, and equally important, to reflect on and express views on the learning process and intended outcomes.

**Figure 1.**
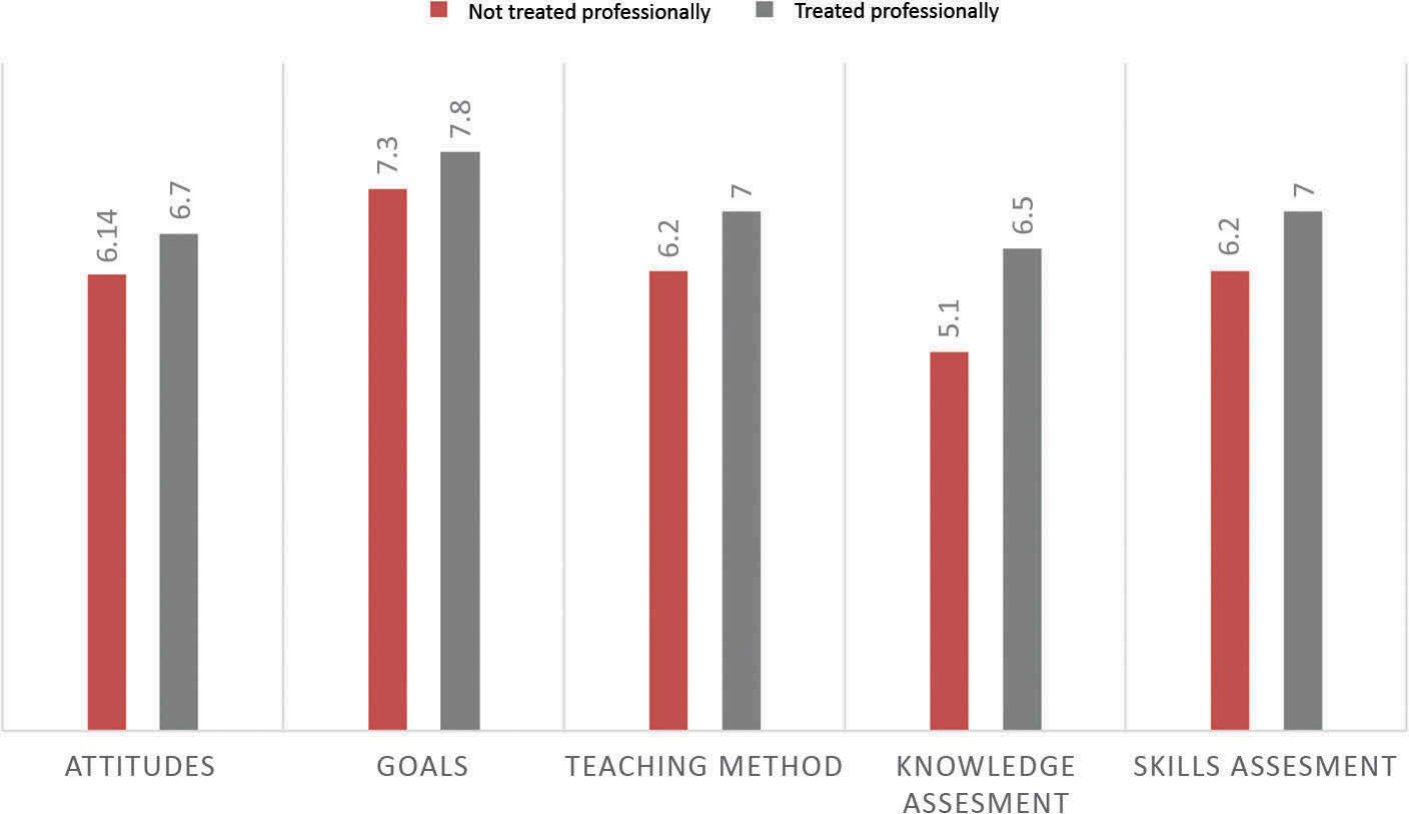
Distribution of studied attitudes who reported being treated in an professional versus unprofessional manner. Among students who claimed being treated in less professional manner, attitudes toward ethics were less favorable (P = 0.001).

In 2013 the population of the United Arab Emirates (UAE) was estimated to be 9.5 million people with 20% being Emirati nationals. This comprises a largely homogenous society with preservation of its unique local culture, which is strongly influenced by Islamic religion. Yet, the cosmopolitan UAE has a unique health system structure with a large community of expatriates as health care providers and educators (trainers), and at the College of Medicine and Health Sciences students (trainees) are principally of native origins. Hence, medical students are tutored and supervised by physicians from diverse cultural, ethnic, and religious backgrounds. Although, a few studies have been conducted elsewhere exploring medical trainees’ attitudes towards the subject of ethics generally [7–9,14–18], to the best of our knowledge, there is no published report from the Middle East exploring students view on professionalism. Little is known about the ethical dilemmas that medical students believe they encounter while working in these environs, or indeed their attitudes and perspectives on goals, learning, and assessment methods for the subject of medical ethics. Hence, this study aimed to examine UAE national medical students’ perceptions of these topics and to identify significant differences of opinion between male and female students. We further aimed to determine if attitudes in these areas were affected by previous experience of ethical conflicts, the presence of positive role models, and personal experiences of being treated in an unprofessional manner.

## Methods

### Survey instrument

After institutional review board (IRB) approval, a paper questionnaire was distributed to medical students with a cover letter indicating the purpose of the study and anonymity of participants. The questionnaire was developed at the University of New Mexico (copyright reserved by Laura Roberts, Cynthia Geppert, and Teddy Warner; permission was obtained from the authors to use the instrument). The survey instrument provides subscales on professionalism and ethics education encompassing 10 domains based on the American Board of Internal Medicine (ABIM) definition of professionalism. The domains include attitudes (25 items), goals [11], learning methods [19], knowledge assessment [6], skills assessment methods [8], educational needs concerning informed consent, principles [9,10], vulnerable populations [20], and relationship and boundaries in clinical practice (31 items) [16]. An additional five questions regarding personal ethics experiences during training and three demographic questions were added. Items in this instrument were rated on a nine point Likert-type scale ranging from 1 ‘strongly disagree’ to 9 ‘strongly agree’. The instrument had been shown to have good reliability with mean retest correlations of r = 0.51, p < 0.05 after seven weeks from first response, and correlations above 0.25 for 54 of the items [16]. This article reports the opinions of graduating medical students’ attitudes toward the subject of ethics, strength of affirmation of goals, and the preferred learning methods, knowledge and skills assessment methods preferences for the topics of ethics and professionalism.

### Subjects

Participants were 108 final year Emirati medical students enrolled at the College of Medicine and Health Sciences, United Arab Emirates University (UAEU). The survey was distributed to students during their surgical subspecialty rotations. The study was conducted with multiple cohorts from August 2009 to February 2013. These students had already completed clinical clerkships in internal medicine, surgery, family medicine, and psychiatry. The survey was distributed at the end of the rotation for each group and collected immediately. The study was conducted among graduating students as we wished to explore their views of experiences regarding the subject of ethics after they had been exposed to the full six-year medical school curriculum, wherein professional attitudes and behavior are expected to have been somewhat imbued. The survey was conducted for five consecutive academic years for the purpose of increasing the sample size as our student numbers annually have been relatively small until recently. All participants had experienced exposure to the same curriculum without any substantial changes over the period of data collection.

### Analysis

Standard descriptive statistics including the mean and standard deviations (SD) of each item were obtained. Associations between variables were assessed using t-tests, and correlation coefficients. Cronbach’s α was used to assess the internal reliability of the questionnaire. Gender differences were assessed using ANOVA tests. All analyses were carried out using SPSS for Windows version 20 (IBM SPSS Inc., Chicago). A *P* value less than 0.05 was considered significant.

## Results

The survey was distributed to a total of 128 (32 male, 96 female) final year students and 108 (24 male, 84 female) completed the survey (84% response rate). Cronbach’s α showed acceptable internal reliability of the questionnaire for section medical students’ attitudes toward ethics and its teaching; students’ ideas about the goal of education in professionalism and medical ethics; medical students’ preferred methods for learning about professional attitudes, values, and ethics (Cronbach’s α 0.649, 0.922, 0.907 respectively).

The mean age of participants was 20.5 ± 8.6 years. Students reported encountering frequent ethical conflicts during training (mean 6.38 ± 1.7, on a scale from ‘1’ never to ‘9’ all the time). Respondents stated that they had received a moderate level of ethics training during medical school (mean 5.14 ± 1.81). They reported that their medical education had helped somewhat in dealing with ethical conflicts (mean 5.39 ± 2.0), and their supervising residents and faculty had been positive role models for ethical and professional behavior (mean = 5.89 ± 1.80). They also reported being usually treated in an ethical and professional manner by supervising residents, faculty, and their training institution (mean 6.19 ± 1.73).

### Attitudes

This section comprised twenty-five statements requesting students to rate each item on a scale of 1–9 (‘1’ being strongly disagree and ‘9’ strongly agree). Negatively phrased questions (i.e., item number 2, 5, 8, 10, 13, 15) were reverse scored when calculating the overall mean of the section. The cohort expressed a positive attitude toward ethics and professionalism (overall mean = 6.51 ± 0.72, range 4.04–8.27) (Table 1). The following items were rated very high by respondents: physicians should possess professionalism (mean = 8.26 ± 1.25); attitudes and values are learned from family, culture, and religion (mean = 7.98 ± 1.22); students face different ethical issues at different points in their training (mean = 7.82 ± 1.31); ethics should be formally taught in the medical school curriculum (mean = 7.35 ± 1.70); and evaluation of students should include assessment of professionalism (mean = 7.43 ± 2.06). The lowest score was for the item ‘attitudes and values are not an appropriate focus for undergraduate medical education’ (mean = 2.75) (Table 1). Cronbach’s α was 0. 649 and increased to 0.668 after excluding negatively phrased items (item 2, 8, 10, 13, and 15) and five items that did not strongly fit this section (i.e., items 19, and 22–25). Reliability was tested by repeating item 2 in the survey, with resultant strong correlation (r = 0.712).

**Table 1.** Medical students’ attitudes toward ethics and its teaching.

	Gender		
(Cronbach’s α 0.649)	Female (N = 84) Mean±SD	Male(N = 24) Mean±SD	Overall (N = 108) Mean±SD
1. Professionalism can be taught and learned.	6.94 ± 1.82	6.96 ± 1.52	6.94 ± 1.75
2. Ethics CANNOT be taught and learned.	3.58 ± 2.40*	2.63 ± 1.66	3.36 ± 2.28
3. Ethics should be formally taught in the medical school curriculum.	7.52 ± 1.48	6.75 ± 2.23	7.35 ± 1.70
4. Attitudes and values are set (fixed, established) by the time students reach residency.	6.27 ± 2.16	5.75 ± 2.40	6.15 ± 2.22
5. There are NO right and wrong answers to ethical issues questions.	6.37 ± 2.08	5.38 ± 2.39	6.15 ± 2.18
6. Ethics is a discipline with its own methods, literature, vocabulary, and content.	7.06 ± 1.68*	5.83 ± 2.65	6.79 ± 1.99
7. Attitudes and values are learned from family, culture, and religion.	7.95 ± 1.29	8.08 ± 0.93	7.98 ± 1.22
8. Attitudes and values are NOT an appropriate focus for undergraduate medical education.	2.71 ± 2.03	2.88 ± 2.25	2.75 ± 2.07
9. Physicians should possess professionalism.	8.31 ± .94	8.08 ± 2.00	8.26 ± 1.25
10. Selection of residents should NOT include assessment of professionalism.	2.96 ± 2.05	3.46 ± 2.21	3.07 ± 2.09
11. Evaluation of students should include assessment of professionalism.	7.36 ± 2.02	7.67 ± 2.20	7.43 ± 2.06
12. Ethical conflicts are common in the everyday practice of medicine.	7.69 ± 1.34*	8.29 ± 0.81	7.82 ± 1.27
13. Training in ethics does NOT help medical students deal with ethical conflicts	3.13 ± 1.94	2.92 ± 1.98	3.08 ± 1.94
14. Students face different ethical issues at different points in their training.	7.91 ± 1.18	7.50 ± 1.67	7.82 ± 1.31
15. Medical training fosters unethical behavior.	4.15 ± 2.25	4.67 ± 2.08	4.26 ± 2.21
16. Medical training fosters professionalism.	7.06 ± 1.75	6.58 ± 1.59	6.95 ± 1.72
17. Medical training fosters cynicism.	4.56 ± 2.22	4.42 ± 2.08	4.52 ± 2.18
18. Students receive adequate training to handle the ethical conflicts they may face.	5.23 ± 2.15	4.92 ± 1.93	5.16 ± 2.10
19. Attention to attitudes, values, and ethical issues helps to prevent cynicism in medical training.	7.01 ± 1.53	6.79 ± 1.96	6.96 ± 1.63
20. It is important that physicians-in-training take an oath or declaration to uphold the values of the profession.	6.98 ± 1.89	6.67 ± 2.46	6.91 ± 2.02
21. Psychiatrists must abide by a different set of ethical guidelines than other physicians.	5.61 ± 2.29	5.25 ± 2.33	5.53 ± 2.29
22. Psychiatrists must abide by a stricter set of ethical guidelines than other physicians.	5.58 ± 2.48	6.50 ± 1.77	5.79 ± 2.36
23. Physicians are more ethical than the general public.	5.59 ± 2.06	5.71 ± 2.22	5.62 ± 2.09
24. Most faculty physicians behave ethically towards students.	5.77 ± 2.03*	4.38 ± 2.55	5.46 ± 2.23
25. Most faculty physicians behave ethically towards patients.	6.62 ± 1.65	6.29 ± 1.99	6.55 ± 1.73
Group means	6.54 ± .65	6.45 ± .90	6.52 ± 0.72

1. Rated on a scale from 1 = ‘much less’ to 5 = ‘same’ to 9 = ‘much more’ attention needed compared to now.

*Statistically significant difference between male and female, P < 0.05

### Goals

This section comprised eleven statements that were rated on a scale of 1–9 (‘1’ strongly disagree and ‘9’ strongly agree). Students expressed positive responses to the goals of the subject of ethics with each individual item mean scores being above seven and an aggregates mean score of 7.63 ± 1.03; range 4.64–9.00. The highest score was for the item ‘goal of ethics is to improve patient care and clinical decision making’ (mean 7.83 ± 1.25) (Table 2).

**Table 2.** Students’ ideas about the goal of education in professionalism and medical ethics.

	Gender		Overall (N = 108)
(Cronbach’s α 0.922)	Female (N = 84) Mean±SD (range)	Male(N = 24) Mean±SD (range)	Male and female Mean±SD (range)
1. To become better people	7.60 ± 1.42	7.50 ± 1.38	7.58 ± 1.41
2. To better recognize ethical issues	7.69 ± 1.27	7.58 ± 1.32	7.66 ± 1.27
3. To develop interpersonal skills useful in resolving ethical conflicts	7.76 ± 1.25	7.50 ± 1.25	7.70 ± 1.25
4. To acquire a working knowledge of social science, philosophy, religion, and law as they apply to clinical care	7.63 ± 1.26*	6.71 ± 1.71	7.42 ± 1.41
5. To improve patient care and clinical decision making	7.93 ± 1.18	7.50 ± 1.45	7.83 ± 1.25
6. To prevent cynicism (negativity) and detachment in interactions with patients	7.57 ± 1.56	7.67 ± 1.27	7.59 ± 1.50
7. To better clarify values-laden (not objective; with personal bias) choices	7.54 ± 1.32*	6.88 ± 1.51	7.39 ± 1.39
8. To reduce the likelihood a physician may make a legal error in the future	7.88 ± 1.14*	6.79 ± 2.36	7.64 ± 1.56
9. To reduce the likelihood that a clinician will face a medical liability suit at some point during practice	7.80 ± 1.03*	6.71 ± 2.27	7.55 ± 1.47
10. To reduce the likelihood that a physician may make an ethical error in the future	7.77 ± 1.12	7.71 ± 1.43	7.76 ± 1.19
11. To learn how to heal our patients in addition to treating them	7.88 ± 1.24	7.63 ± 1.53	7.82 ± 1.31
Group means	7.73 ± .99	7.29 ± 1.11	7.63 ± 1.03

1. Rated on a scale from 1 = ‘much less’ to 5 = ‘same’ to 9 = ‘much more’ attention needed compared to now.

*Statistically significant difference between male and female, P < 0.05

### Learning methods

This section comprised nineteen statements that students were asked to rate as to which specific methods of education and training should be included in the curriculum. Students endorsed all teaching and learning methods and the most strongly accepted methods were interactions with patients in routine training situations (mean = 7.63 ± 1.28), followed by positive role models of ethical and professional behavior (mean = 7.45 ± 1.52), and incorporation of ethical issues into teaching rounds (mean = 7.28 ± 1.53) (Table 3).

**Table 3.** Medical students’ preferred methods for learning about professional attitudes, values, and ethics (Mean±SD).

	Gender		Overall (N = 108)
(Cronbach’s α 0.907)	Female (N = 84) Mean±SD (range)	Male(N = 24) Mean±SD (range)	Male and female Mean±SD (range)
1. Case conferences (presentations)	6.98 ± 1.75*	6.08 ± 1.98	6.78 ± 1.83
2. Grand round presentations	7.19 ± 1.49*	6.08 ± 1.67	6.94 ± 1.59
3. Lectures	6.52 ± 1.78	6.00 ± 2.06	6.41 ± 1.85
4. Clinical rounds	7.54 ± 1.35*	6.37 ± 1.789	7.28 ± 1.53
5. Discussion groups of peers led by a knowledgeable clinician	7.88 ± 1.17	7.54 ± 1.32	7.81 ± 1.20
6. Discussion groups of peers without leadership by a clinician	5.49 ± 2.07	5.83 ± 1.71	5.56 ± 2.00
7. Watching videotapes on ethics topics followed by discussion led by a knowledgeable clinician	6.43 ± 1.85	6.33 ± 2.30	6.41 ± 1.95
8. Incorporation of ethical issues into lectures and teaching rounds	7.62 ± 1.24*	6.79 ± 1.62	7.44 ± 1.37
9. Role modeling of ethical reasoning and behavior by faculty	7.48 ± 1.34	7.37 ± 2.06	7.45 ± 1.52
10. Interactions with standardized (e.g., simulated) patients	6.96 ± 1.48	6.62 ± 1.69	6.89 ± 1.52
11. Interactions with patients in routine training situations	7.76 ± 1.16	7.17 ± 1.69	7.63 ± 1.28
12. Independent reading	5.76 ± 2.13	5.87 ± 1.78	5.79 ± 2.05
13. Web-based educational approaches	5.79 ± 2.100	6.17 ± 1.373	5.87 ± 1.96
14. Directed (assigned) reading with tutorial discussions	6.27 ± 1.835	5.71 ± 2.293	6.14 ± 1.95
15. Directed ethics research with a mentor	6.32 ± 1.977	5.67 ± 2.200	6.18 ± 2.04
16. Discussion of clinical ethics with ethics consultants	7.19 ± 1.596	6.83 ± 2.078	7.11 ± 1.71
17. Discussion of the legal aspects of patient care with attorneys (lawyers, legal experts)	7.16 ± 1.604	6.42 ± 1.692	6.99 ± 1.65
18. Discussion of the spiritual (religious) aspects of patient care with religious leaders	7.31 ± 1.448*	6.04 ± 1.922	7.03 ± 1.64
19. Discussion of the cultural aspects of patient care with cultural experts	7.15 ± 1.477*	6.29 ± 1.829	6.96 ± 1.59
Group means	6.88 ± .99	6.38 ± 1.19	6.77 ± 1.06

1. Rated on a scale from 1 = ‘much less’ to 5 = ‘same’ to 9 = ‘much more’ attention needed compared to now.

*Statistically significant difference between male and female, P < 0.05

### Knowledge assessment

This section comprised six items on preferred methods for ethics assessment. The highest ranked methods were: attending staff observation during clinical supervision (mean = 7.63 ± 1.44), using standardized (e.g., simulated) patient interactions (mean = 6.82 ± 2.12) followed by oral examinations (mean = 6.22 ± 2.47), and less favored were multiple choice examinations (mean = 5.19 ± 2.56), short-answer questions (mean = 5.38 ± 2.59), and essays (mean = 5.10 ± 2.50) as assessment methods for the subject of ethics (Table 4).

**Table 4.** Students’ views of appropriate needed methods for assessing knowledge of professional attitudes, values, and ethics.

	Gender		
(Cronbach’s α 0.837)	Female (N = 84) Mean±SD (range)	Male(N = 24) Mean±SD (range)	Overall (N = 108) Mean±SD (range)
1. Multiple choice examinations	5.19 ± 2.66	5.17 ± 2.22	5.19 ± 2.56
2. Short answer questions	5.35 ± 2.68	5.50 ± 2.28	5.38 ± 2.59
3. Essays	5.11 ± 2.53	5.08 ± 2.47	5.10 ± 2.50
4. Oral examinations	6.43 ± 2.42	5.50 ± 2.55	6.22 ± 2.47
5. Standardized (e.g., simulated) patient interactions	6.96 ± 1.98	6.33 ± 2.55	6.82 ± 2.12
6. Clinical supervision	7.67 ± 1.50	7.46 ± 1.25	7.63 ± 1.44
Group means	6.11 ± 1.71	5.84 ± 1.73	6.05 ± 1.71

1. Rated on a scale from 1 = ‘much less’ to 5 = ‘same’ to 9 = ‘much more’ attention needed compared to now.

### Skills assessment

This section comprised eight statements concerning assessment methods for ethics skills. The highest scores for preferred assessment methods were direct faculty observation of students’ interactions with actual patients (mean = 7.62 ± 1.26), faculty observation of students’ interactions with clinical team members (mean = 7.31 ± 1.46), and patients evaluation of students (mean = 7.19 ± 2.20) (Table 5).

**Table 5.** Students’ views of appropriate needed methods for assessing skills of professional attitudes, values, and ethics.

	Gender		Overall (N = 108)
(Cronbach’s α 0.845)	Female (N = 84)	Male (N = 24)	Male and female
1. Standardized (e.g. simulated) patients’ assessment of their interactions with students	6.84 ± 1.70	7.04 ± 1.68	6.89 ± 1.69
2. Faculty direct observation of students’ interactions with actual patients	7.60 ± 1.31	7.67 ± 1.13	7.62 ± 1.26
3. Faculty observation of videotaped interactions of students with actual patients	6.45 ± 2.04	6.46 ± 2.13	6.45 ± 2.05
4. Faculty observation of students’ interactions with clinical team members	7.31 ± 1.51	7.29 ± 1.33	7.31 ± 1.46
5. Students’ written and observational skills in analyzing ‘trigger’ videotapes	5.77 ± 2.14	5.92 ± 1.56	5.80 ± 2.02
6. Written exercises as follow-ups to standardized (e.g., simulated) patient interactions	5.73 ± 1.95	6.00 ± 2.00	5.79 ± 1.95
7. Evaluation of students by non-faculty staff	6.54 ± 1.98	6.33 ± 2.60	6.50 ± 2.12
8. Evaluation of students by patients	7.20 ± 1.99	7.13 ± 2.86	7.19 ± 2.20
Group means	6.68 ± 1.31	6.73 ± 1.28	6.69 ± 1.30

1. Rated on a scale from 1 = ‘much less’ to 5 = ‘same’ to 9 = ‘much more’ attention needed compared to now.

### Associations between different domains

Students’ responses showed a positive correlation between the extent of ethics education and its usefulness in dealing with ethical conflicts (r = 0.642, P < 0.000). There was a correlation between encountering ethical conflicts during training and the attitudes towards the subject of ethics (aggregates score r = 0.225, P < 0.05). There was a correlation between being treated in an ethical and professional manner and endorsement of all the listed goals for the subject of ethics (aggregate score r = 0.228, P < 0.05), methods of teaching and knowledge assessment (aggregates score r = 0.412, P < 0.01), (r = 0.427, P < 0.01) respectively.

Further analysis was conducted by stratifying the students into two categories, those scoring lower than 5.5 (29 students) for the item ‘being treated in an ethical and professional manner’ and those scoring more than 5.5 (65 students). This analysis revealed a significant difference between the two groups as to their attitudes toward the subject of ethics (mean 6.14 ± 0.83 vs. 6.68 ± 0.60, P = 0.001), affirmation of the goals of ethics education (7.29 ± 1.10 versus 7.78 ± 0.96, p = 0.022), their endorsement for teaching methods (6.22 ± 1.05 vs. 7.02 ± .97, p = 0.00), and their preferred assessment methods (6.46 ± 1.46 vs. 6.05 ± 1.71, p = 0.00), all in favor of students who believed that they were treated in an ethical and professional manner.

### Gender differences

When compared to female students, considerably more male students recognized that ethics is a discipline with its own methods, literature, vocabulary, and content (p = 0.040); and agreed that ethical conflicts are common in everyday practice of medicine (p = 0.008). Significantly more females believed that most faculty physicians behave ethically towards students (p = 0.019). Despite similar curricular exposure for both genders, when asked about how much training in ethics have you received to date during medical school, responses differed (male mean = 6.04 ± 1.08, female mean = 4.88 ± 1.89, p = < 0.05).

Significantly more females were in favor of grand round presentations (p = 0.002), clinical rounds (p = 0.005), incorporation of ethical issues into lectures and teaching rounds (p = 0.008), and discussion of the spiritual aspects of patient care with religious leaders (p = 0.005) as teaching methods for the subject of ethics.

A statistically significant difference was also detected for the item: ‘during your medical training, how often have you been treated in an ethical and professional manner by your supervising residents, faculty, and the training institution where male students more frequently claimed that they were being treated in an unprofessional manner by supervising residents, faculty, and their training institution (male mean = 5.54, female mean = 6.37, p = 0.038). Students who reported being treated in an unprofessional manner registered a lower aggregates score for the domain of attitudes toward ethics as a subject (mean 6.14 ± 0.83 vs. 6.68 ± 0.60, p = 0.001) and gave less endorsement of the subject goals (7.29 ± 1.10 vs. 7.78 ± 0.96, p = 0.022) (Figure 1).

## Discussion

The results of this study affirm positive attitudes towards ethics education by final-year medical students from a rapidly developing country in the Arab Middle-East region. These results compare favorably with others examining medical students’ attitudes towards the subject of ethics and identifying the need for its teaching [7,9,16–19,21]. Also consistent with earlier reports [19,22], students recorded encountering ethical conflicts moderately frequently during training, and believed they had received a reasonable level of ethics training during medical school. They also reported that their overall medical education had helped somewhat to deal with ethical conflicts.

In our setting, ethics and professionalism are taught in an integrated fashion through didactic sessions, small group supervision, and special activities (a ‘White Coat’ ceremony, debate and orientation sessions). We estimate that medical students receive on average 40 hours of formal instruction on ethics and professionalism-related topics, mostly in pre-clinical years. However, it is difficult to quantify the amount of personal experience and clinical supervision focusing on medical ethics during clinical clerkships. As Howe [23] reported over two decades ago, students’ levels of satisfaction with medical ethics teaching was directly related to the teaching lauds they had received.

Despite a lack of consensus in the literature as to the best method for teaching professionalism in medicine, in a recent best evidence medical education systematic review, it has been reported that professionalism is learned most effectively through role models and mentoring guided by faculty [5]. Consistent with the findings of prior surveys this cohort valued diverse learning approaches for ethics [9,16]; they expressed a strong preference for interactive methods of learning, such as discussion groups of peers led by a knowledgeable clinician, and interactions with patients during routine training with strong endorsement of the value of positive role models as the preferred learning method. There has been a call in the region for adoption of more interactive and engaging methods for ethics teaching instead of the current tendency which has a focus as lecturing [24]. Students in this survey also reaffirmed the viewpoint that small group teaching is an effective strategy for learning more about medical ethics, being superior to lectures for developing moral reasoning skills and getting familiar with professional values [20,25–27]. We support the view of Dennis and Hall 1977, whom suggested diverse and continuing exposure to the moral dimensions of medical care [28]. This is especially notable given the results of an earlier study that indicated a statistically significant increase in the level of moral reasoning of students exposed to a medical ethics course compared with a control group [21]. Furthermore, our students’ beliefs on how ethics could be assessed were comparable to the findings of prior surveys of medical students [16], wherein skills assessment was favored over knowledge assessment, with clear preferences for direct faculty observation of students’ interactions with actual patients and medical team members. This may be explained by the observation that our students viewed ethics as a practical subject, and its learning and assessment need to be an integral part of routine clinical care.

In the present study, female students endorsed the goals of ethics education more strongly than their male counterparts. Furthermore, with regard to teaching and learning of ethics in clinical practice, significantly more female than male students approved case presentations, grand round presentations, clinical rounds, incorporation of ethical issues into lectures, and teaching rounds, discussion of the spiritual aspects of patient care with religious leaders, and discussion of the cultural aspects of patient care with cultural experts. The cause of these gender differences is unknown, but maybe related to differences in learning behavior preferences between genders [24]. However, caution should be observed in the interpretation of these gender differences as the number of male respondents was smaller. Although opportunities for entry to medical school for both men and women is similar at our college, fewer male applicants are admitted each year (approximately 25% versus 75% female), which may be attributed to wider career choices available to men, influences of family commitments, and in particular special cultural values. However, in terms of the goal of application of ethical principles that these preferred learning methods afford, the gender differences reported here may be supported by the observations of Gilligan, Gilligan & Attanucci [29,30] in their research indicating that females tend to approach ethical dilemmas in a contextualized, narrative way that looks for resolution of problems through attention to details of the particular situation while men tended to apply more general abstract principles without attention to the unique circumstances of the case. Studies indicate that women resident physicians perceive a greater need for ethics preparation, value it more and see benefit in a more diverse set of educational methods than do men [31–33]. As to whether there are inherent differences in female perceptions as suggested in prior surveys of medical students [9,24,34] we found that despite similar curricular exposure, more women believed that the level of ethics training they received was moderate compared to men (mean male 6.04, mean female 4.88). Our observations are consistent with Bickel and Ruffin, who documented that more female medical students reported inadequate curricular coverage of many subjects compared with males [14]. Similarly, Roberts et al. have reported women’s desire for more education regarding ethical dilemmas compared with their male counterparts [17]. This could reflect the fact that females are looking for greater exposure to ethical issues and hence value this subject more than their male counterparts [14]. Also despite the expectation that in an outcomes-based curriculum, formal ethics education would be comparable for male and female students, it is plausible that personal experiences and the clinical exposure might be different.

Male students believed that they were more frequently treated in an unprofessional manner by supervising residents and faculty compared to their female counterparts. This is in contrast to medical school graduation questionnaire findings from the Association of American Medical Colleges wherein more graduating female students revealed being publicly belittled or reported incident of mistreatment [14]. A study at University of Toronto School of Medicine also revealed that more women (46%) than men students (19%) reports sexual harassment [35]. The cause of the differences between our findings and others is unknown; however, it may be hypothesized that in conservative societies with special cultural circumstances and religious influences, women might be advantaged, as major social and professional consequences may follow misconduct towards women, especially if of a sexual nature. It is also possible that male students learning in small groups perceive and apply more scrutiny and emphasis on professional and ethical attitudes and behavior of themselves, peers and teachers. Analysis of future written justifications may however help clarify the reasoning behind responses to this component of the questionnaire.

Previously, no significant correlation between attitudes towards ethics and religious background has been identified [7]. Furthermore, it has been reported that medical students with religious studies in their educational history rate the study of ethics to be less important to good medical care than their peers who have no religious studies background [8]. In contrast, we believe that in our context, religious moral principles are influential and serve as a guide for conduct in medical settings. This might also account for this relatively young group (mean 20.5 ± 8.6 years) of students being receptive to ethical considerations despite perceived limited exposure in the curriculum. A longitudinal study may assess the validity of these findings and determine how religious beliefs, personal histories, gender, social and cultural backgrounds affect ethical practice in clinical decision making of medical students and qualified practitioners.

Interestingly, students who responded with low scores on the item ‘being treated in an unprofessional manner’ also scored low on the domain ‘attitudes towards the subject of ethics’. One explanation may be that these students had developed a negative perception toward ethics as a subject and are consequently more cynical due to personal experiences, either during medical education and training environments or even prior to medical school enrollment. It has been suggested that compassion may be eroded in the course of medical training and be replaced by negative attitudes [36–38]. This warrants the attention of educators and further exploration.

A limitation of this study is that students’ views were solicited at a single point in time and at one medical school. The sample included a cohesive group with similar exposure to ethics learning which made it difficult to assess changes in students’ attitudes during exposure to medical ethics courses. However, the study’s strength derives from identifying student perspectives about ethics and professionalism in a region where documented studies are scant or non-existent. This information may also help in the development of curricular content and methods that are more acceptable to trainees, as has been previously suggested [31,39]. The medical education environment has become more complex in terms of challenging ethical dilemmas, with students grappling with the everyday challenges of diversity in clinical ethics and what some authors regard as the twin pitfalls of moral relativism and moral imperialism [40–42]. Medical students often perceive that curricula and support from faculty insufficiently addresses these issues that contribute to their ability to become culturally competent practitioners. The literature nevertheless indicates that well-developed educational interventions can enhance learning of moral reasoning, medical professionalism, and ethics. Understanding the perspectives of medical students on these topics can contribute significantly to how the pedagogy, if not core ethical principles, are contextualized. The realities of diversity in the field at the educational and philosophical levels should not be underestimated and awaits further investigation in our context.
